# Methods for Studying Mucociliary Transport

**DOI:** 10.1016/S1808-8694(15)30133-6

**Published:** 2015-10-19

**Authors:** Sergio Henrique Kiemle Trindade, João Ferreira de Mello Júnior, Olavo de Godoy Mion, Geraldo Lorenzi-Filho, Mariângela Macchione, Eliane Tigre Guimarães, Paulo Hilário Nascimento Saldiva

**Affiliations:** a PhD student, Otorhinolaryngology Department – University of São Paulo Medical School. Attending ENT - Hospital do Servidor Público Estadual de São Paulo e Hospital Estadual de Bauru; b Associate Professor – University of São Paulo Medical School Department of Otorhinolaryngology – Head of the Allergy Group in Otorhinolaryngology; c PhD in Otorhinolaryngology - University of São Paulo Medical School – Professor – University of São Paulo Medical School Department of Otorhinolaryngology - FMUSP, Member of the Allergy Group; d PhD. Department of Cardio-Pneumology – Discipline of Pneumology – São Paulo University Medical School – Professor at the Department of Pneumology – University of São Paulo Medical School - FMUSP; e PhD, Researcher – Department of Experimental Air Pollution, LIM-05 – University of São Paulo Medical School; f PhD, Researcher – Department of Experimental Air Pollution, LIM-05 – University of São Paulo Medical School; g Full Professor of Pathology – University of São Paulo Medical School - FMUSP. Study carried out at the Otorhinolaryngology Department – University of São Paulo Medical School and Laboratory of Experimental Air Pollution – Department of Pneumology – University of São Paulo Medical School, FMUSP

**Keywords:** rheological analysis, ciliary dyskinesia, ciliary beating frequency, mucociliary transport

## Abstract

Mucociliary transport dysfunctions can impair the quality of life of patients suffering from chronic rhinossinusitis and lead to severe consequences such as alterations in respiratory physiology or even death as in cases of cystic fibrosis and primary ciliary dyskinesia. Therefore, it is crucial to understand the physiology of the mucociliary apparatus and how its components (cilia, mucus-periciliary layer and its interaction) affect the clearance of respiratory secretions. **Aims:** This paper aims to review and to discuss different techniques for studying mucociliary transport and their clinical and experimental applicability. **Conclusions:** The methods listed in this revision provide us with valuable information about different aspects of the mucociliary transport. Some of the methods listed are more suitable for clinical practice and present reproducible results. Others, show only applicability in experimental settings due to technical difficulties or financial limitations. However, it is important to emphasize that up to now there is no method that can evaluate ciliary beating frequency (CBF) in vivo and in situ. Such a method would become a valuable tool in the scientific scenario and in the clinical practice, supporting the diagnosis of ciliary dyskinesias and avoiding the use of invasive procedures to corroborate the clinical suspicion

## INTRODUCTION

The respiratory tract is constantly exposed to different types of noxious agents, which enter in direct contact with it’s mucosa, such as microorganisms and air pollutants. For homeostasis maintenance of such delicate and complex system, the respiratory tract counts on a sophisticated mechanism of defense: “The mucociliary aparatus”^1^.

The airway mucosa, from the nasal cavities all the way to the respiratory bronchia, is made up of a pseudostratified, cylindrical ciliated epithelium, intertwined with submucosal glands and goblet cells, which are the cell elements responsible for the production of mucus. In the nasal cavity, seromucous glands are located in the submucosa and are the ones responsible for mucus production. In the paranasal sinuses, goblet cells prevail^2^.

The mucociliary apparatus, as its main function, removes potentially aggressive particles and substances from the respiratory tract, by means of cilia transportation or, alternatively, by cough and sneezing, in cases of mucus overproduction such as allergic rhinitis, chronic bronchitis, cystic fibrosis and asthma^1^.

The cilia are the thrusters of the mucociliary transportation.

The number of cilia per cell varies between 50 and 100, being influenced by age and their position in the respiratory tract. They are made up of a classic axoneme with nine pairs of peripheral microtubules (A) and one central (B). The peripheral pairs are connected to the neighboring pairs by two arms of dynein, one inside and one outside, and also connected to the central pair by means of contractile proteins. The connection between microtubules A and B, through the internal and external arms, ATP-mediated, causes cilia shifting and their consequent beating. Under normal conditions, the cilia from the septal mucosa and the lower turbinates beat at a frequency of 12-15 Hz (beats/second). Ciliary beat has a coordinated sequence, producing a metachronic wave, of which mechanism of control remains unknown. This metachronic wave guides the mucus flow in the nasal cavities towards the nasopharynx, later on to the oropharynx and hypopharynx, from where the secretions are swallowed^3^.

Ultra-structural alterations in the cilia, such as the lack of internal or external dynein arms, or peripheral or central microtubule pairs transposition or deletion, produce significant changes in the frequency and pattern of ciliary beating, which cause secretion stasis and repetitive respiratory infections, such as primary ciliary dyskenisias.^4^.

Cilliary beat is highly influenced and dependant on viscoelastic properties and respiratory mucous transportation. The “carpet” that lines the ciliary epithelium is made up of respiratory mucous and periciliary fluid. The mucus has two layers: aqueous phase below and the viscous phase above, with a thickness that varies between 0.5 and 2.0µm. The periciliary fluid is basically made of water, which concentration is determined by the active Na and Cl transportation through the respiratory epithelium. Cilia beating in its initial phase has the action component, in which the cilium goes through he aqueous phase easily; and later there is an interaction with the viscous phase at the cilium tip, where the mechanical stimulus happens and moves the mucus layer. In the recovery phase, the cilium bends laterally, returning to its original position by the aqueous phase in order to restart a new cycle of ciliary beating1. Notwithstanding, there is evidence in the literature that cilia beating may simply happen from anterior to posterior, contrary to the theory that the cilia bend laterally in the recovery phase4. The mucus carpet renews itself every 20 to 30 minutes, with an average velocity of 6mm/min, however, these values vary much, even among normal individuals^3^.

The nasal mucus is a complex mixture of secretions coming from the goblet cells, the submucosal glands, tear glands and water. It also has inflammatory cells such as macrophages, basophile, mast cells and eosinophils, which concentrations vary according to disease stage^3^. The nasal mucus also has proteins such as albumin, lactoferrin, glycoproteins (high molecular weight mucins), lysozymes and all the major imunoglobulins. Secretory IgA and IgG make up 50% of the total protein content in the nasal mucus. Other substances such as electrolytes, cytokines, interferons, leukotrienes, histamine, P substance and bradykinin are also part of the nasal mucus. Under pathological conditions, there is an intense change in mucus composition, directly and indirectly affecting the mucociliary function, especially because of alterations in the respiratory mucus viscoelastic properties^3^.

The main control mechanism of nasal secretion is autonomic, and parasympathetic stimulation increase secretion volume. Although this mechanism is basically mediated by acetylcholine, the vasoactive intestinal peptide (VIP) suggests the existence of mechanisms which are not mediated by acetylcholine^3^.

Because of the importance mucociliary movement alterations have in the airway physiology, it is fundamental to develop and use methods for experimental and clinical application in the three major mucociliary movement components:

1) mucociliary beating frequency and pattern,
2) physical and motility properties of the respiratory mucus,
3) interaction between cilia and overlapping layer.

## LITERATURE REVIEW

### Methods to study the frequency and pattern of cilliary movement

Video-endoscopic technique

It is a modification of the technique initially described by Braga et al.5, in which the goal is to check ciliary beat frequency (CBF) in ex vivo experimental preparations. With a light microscope, under 100x magnification, connected to a video camera and a video monitor, one should focus on the group of ciliary cells under study. A strobe light is placed in front of the ciliated epithelium, emitting light flashes at a rate that varies between 0 and 30 Hz. The light emitted by the source is reflected by the ciliary epithelium and by the thin mucus layer that coats it. This reflection is cyclic, because of changes in cilia direction. By manual control, it is possible to define CBF when the flash triggering sequence is identical to that of CBF, and one can no longer see movement on the epithelium surface.

The video-endoscopic approach is useful in ex vivo preparations, such as frog palate, or fragments of surgical specimens from the respiratory mucosa of human beings. The use strobe light in bronchoscopes to analyze CBF in vivo has been attempted by some authors6, however, they were of little clinical applicability because of technical difficulties. One of its disadvantages is the subjective evaluation of the CBF.

### Video microscopy

The ex vivo CBF analysis may also be carried out through the evaluation of light microscopy recordings of ciliated epithelium fragments, with high speed video cameras (Digital High Speed Video - DSHV), which help obtain hundreds of frames per second. Chilvers et al.^4^ developed CBF measures with the help of a camera able to capture 400 frames per second, allowing one to view the epithelium from above, anterior and laterally, contrary to the methods previously developed, which allowed only an upper view of the epithelium. Through this method they were able not only to measure CBF, but also to check the beating pattern under different alterations of the cilliary axoneme in primary dyskinesias. It is the most reliable method for CBF analysis, since in disorders such as the transposition of microtubule pairs, the cilia have normal CBF, however with inefficient circular beating pattern for secretion clearance, which could be ignored by videoscopic methods.

### Mucus study - Nasal mucus collection

The first difficulty in studying respiratory mucus is the lack of simple and not very invasive methods that helps in collecting enough respiratory secretion. We may obtain mucus samples from the nasal cavity by using tracheal aspiration tubes, carefully positioned on the floor or near the lateral wall. We must use the minimum vacuum possible, which helps in collecting the samples with little mucus stirring, in an attempt to avoid protein denaturation and the incorporation of air bubbles^7^. We may also use cytology brushes, which have the disadvantage of collecting little amounts of mucus^8^.

Samples may also be collected from a patient under general anesthesia, before carrying out surgical procedures, in such cases it is possible to collect larger amounts of mucus without bringing discomfort to patients. It is necessary to preclude the use of anticholinergic medication such as atropine, which alter respiratory mucus properties.

## PHYSICAL PROPERTIES OF THE MUCUS

### Rheology

**Figure 1:**
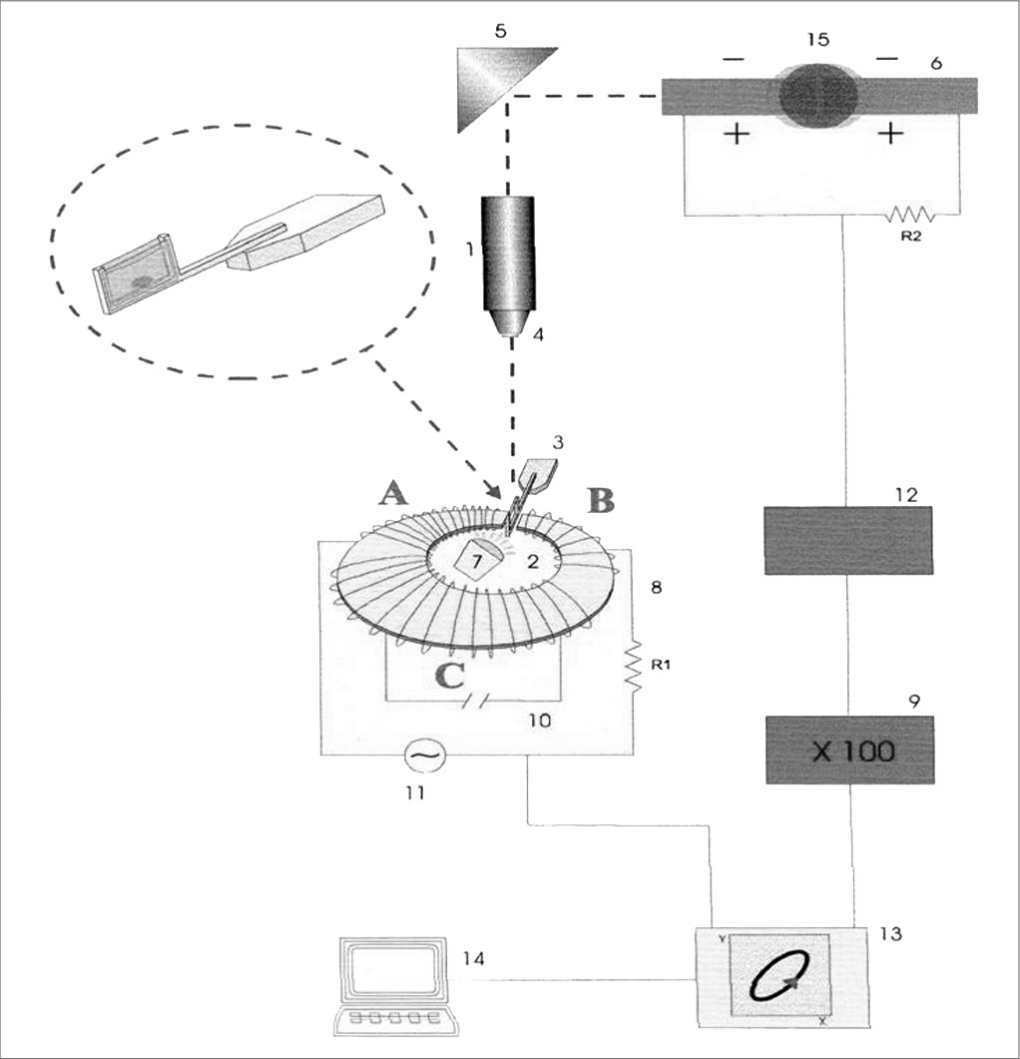
Schematic drawing of the magnetic microrheometer. 1) Light source, 2) Thoroid, 3) Specimen holder, 4) Microscope lens, 5) Set of mirrors, 6) Pair of photocells, 9) Amplifier, 13) Oscilloscope, 14) Microcomputer, 15) Moving steel microsphere. Source: Nakagawa et al., 2000.

The respiratory mucus is a non-Newtonian fluid and its mechanical properties change according to the intensity and frequency of the force applied. Thus, it is paramount to analyze what impact stimulus frequency has on the mucus samples. Low frequency stimuli (1 and 10 radians/second) reflect how the mucus react as it is transported by the cilia; and high frequency stimuli (100 radians/second) simulate the stimulus by cough and sneezing. The magnetic micro-rheometer described by King and Macklem^9^ and modified by Silveira et al.^10^ is an elegant method developed to analyze microsamples of mucus (Figure 1). It is based on measuring the shifting of a small steel ball immersed in a mucus sample, which moves under the influence of an electromagnetic field of sine wave oscillatory pattern. The mucus sample is placed in a glass specimen holder, which is placed in a gap in the micro-rheometer electromagnetic thoroid, assembled in a projection microscope platform. The moving steel ball shadow is projected on a pair of photocells, giving off an electrical signal which is proportional to the ball’s movement. The electrical signal of the thoroid’s alternate current and those from the photocells pair are captured by a digital oscilloscope and analyzed off-line by an IBM compatible microcomputer^1^.

The moving steel ball inserted in the mucus sample acts as a rheologic probe, since its movements are counteracted by elastic and viscous forces. By determining the time lapse between the force application and the resulting deformity, it is possible to completely decompose the system’s impedance in elastic and viscous components separately.

The results from the rheologic analysis are expressed in logG*, which represents the vectorial summation of viscosity and elasticity, reflecting the complete impedance of the system and tandel, representing the ratio between viscosity and elasticity. G* and tandel affect differently the mucus transportation. In general lines, sample transportability reduced with the increase in logG* (total impedance)^11^, ^12^, ^13^. There is an optimal value for tandel, as attested by Puchelle et al.^14^, where the secretion transportation is carried out in a more optimized fashion.

### Adhesiveness and Hydrophobicity

Besides the rheologic properties, other characteristics such as adhesiveness and hydrophobicity, represent fundamental physical properties for mucus transportation, especially because of the high frequency stimuli, such as cough and sneezing.

Adhesiveness characterizes the attraction forces between one adherent surface and one adhesive system1. In the respiratory system, mucociliary transportation involves the interaction of the respiratory epithelium surface and the mucus layer, and during a cough spell these attraction forces added to the interface forces between the liquid and gel phases in the respiratory mucus, control the efficacy of the cough-mediated transportation. In other words, adhesiveness corresponds to the force necessary to separate the adhesive fluid (mucus) and the adherent surface (respiratory epithelium).

Hydrophobicity characterizes the capacity a fluid has of spreading over a flat solid and smooth surface without electrostatic charge. Such phenomenon occurs due to a finite interaction between the solid surface and the fluid molecules. The degree of hydrophobicity can be determined by the value of the “contact angle” between the liquid/air interface and the horizontal plane^14^.

**Figure 2:**
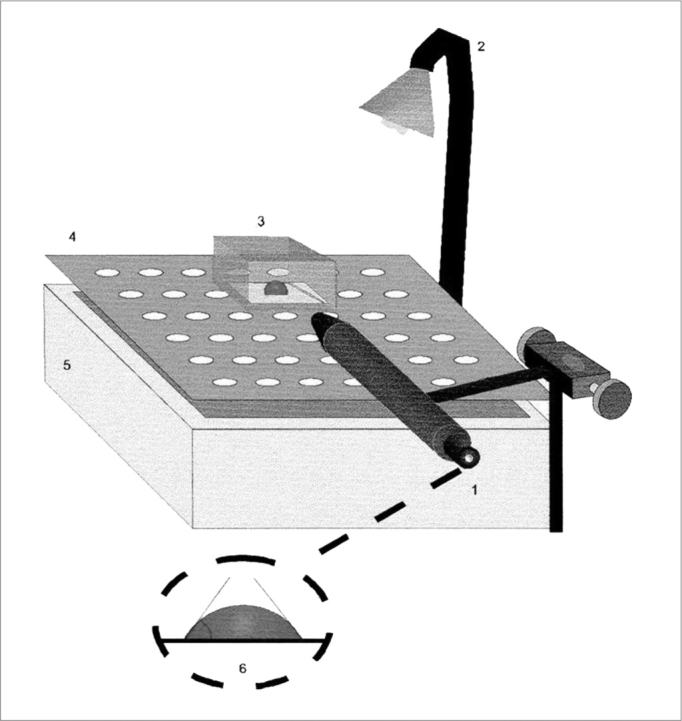
Schematic representation of the contact angle measurement. 1) Magnifying glass coupled to the goniometer, 2) Light source, 3) Acrylic chamber, 4) Cleft platform, 5) Water reservoir, 6) Contact angle measurement representation. Source: Nakagawa et al., 2000.

The contact angle is seen by means of a 25x magnifying glass, coupled to a goniometer with a 0 to 180º angular scale (Figure 2). The measuring surface is treated with sulphochromic acid, which removes the electrostatic charges and the system is kept moist with water vapor at 37º C.

Mucus sample with low contact angle tend to be better transported by cough^15^.

### Analysis of the mucus’s biochemical composition

The respiratory mucus’s biochemical composition may significantly alter its viscoelastic characteristics, and under normal conditions, the body tends to produce a mucus with optimum viscoelasticity to be moved^16^.

The concentration of mucins or glycoproteins in the mucus, such as fucose, produced by the submucosal glands and goblet cells, and IgG derived in part from local production and the other part from serum production, seems to be the major determining factor for both viscosity and elasticity of the samples. They correspond to approximately 73% of the variation of rheologic parameters in chronic rhinosinusitis. In chronic bronchitis and cystic fibrosis, the main factors responsible for an increase in viscoelasticity are the DNA derived from neutrophils and Actin-F. Although we do not know the exact mechanisms through which IgG increases viscoelasticity, controlling inflammatory processes reduces viscoelasticity in parallel to the drop in IgG levels in the mucus^17^.

The glycoproteins in the mucus are formed by end-to-end associations, linked by disulphide bonds, making them very large macro-molecules. Other intermolecular bonds such as ionic bonds, hydrogen bonds and the Van der Waal’s forces contribute to the creation of glycoprotein networks, which also participate in determining the viscoelastic properties of the respiratory mucus. DNA molecules may also be linked to glycoproteins through these bonds, making viscoelasticity to be determined not only by the quantity of glycoproteins, but also by other structures linked to it and through different intermolecular bonds^18^.

The quantification of glycoproteins and their subtypes, as described by Majima et al.18, may be carried out by means of radioimmunoassay (ELISA), when they developed specific antibodies for each subtype of glycoproteins, deriving from the different mucus producing cells.

### Transepithelial electrical potential difference (PD)

The active transportation of ions through the respiratory epithelium plays an important role in the composition and volume of the periciliary fluid. Water secretion and absorption through the respiratory epithelium happen in a passive way through diffusion, in response to local osmotic gradients created by active ion transportation. Cl secretion and Na absorption create a difference in electrical potential between the epithelium surface and the submucosa, and under certain conditions it may shift the fluid to within the airway lumen, or towards the interstitium, respectively. Active ion secretion acts basically by regulating the height and composition of the periciliary fluid, allowing the cilliary beat to reach its most effectiveness in transporting secretions. The difference in electrical potential between the mucosa and submucosa surfaces represents the results of transepithelial ion transportation. PD reductions can represent alterations in this transport or a loss of epithelium integrity^19^.

Experimentally, the measure of transepithelial electrical potential difference may be determined by using a preparation of a dog trachea, in which an electrode placed on the submucosa and another placed on the epithelial surface measure the difference in potential, which is usually around 30 mV (negative lumen)^20^.

### Ex vivo analysis of mucociliary transport - Frog palate preparation

**Figure 3:**
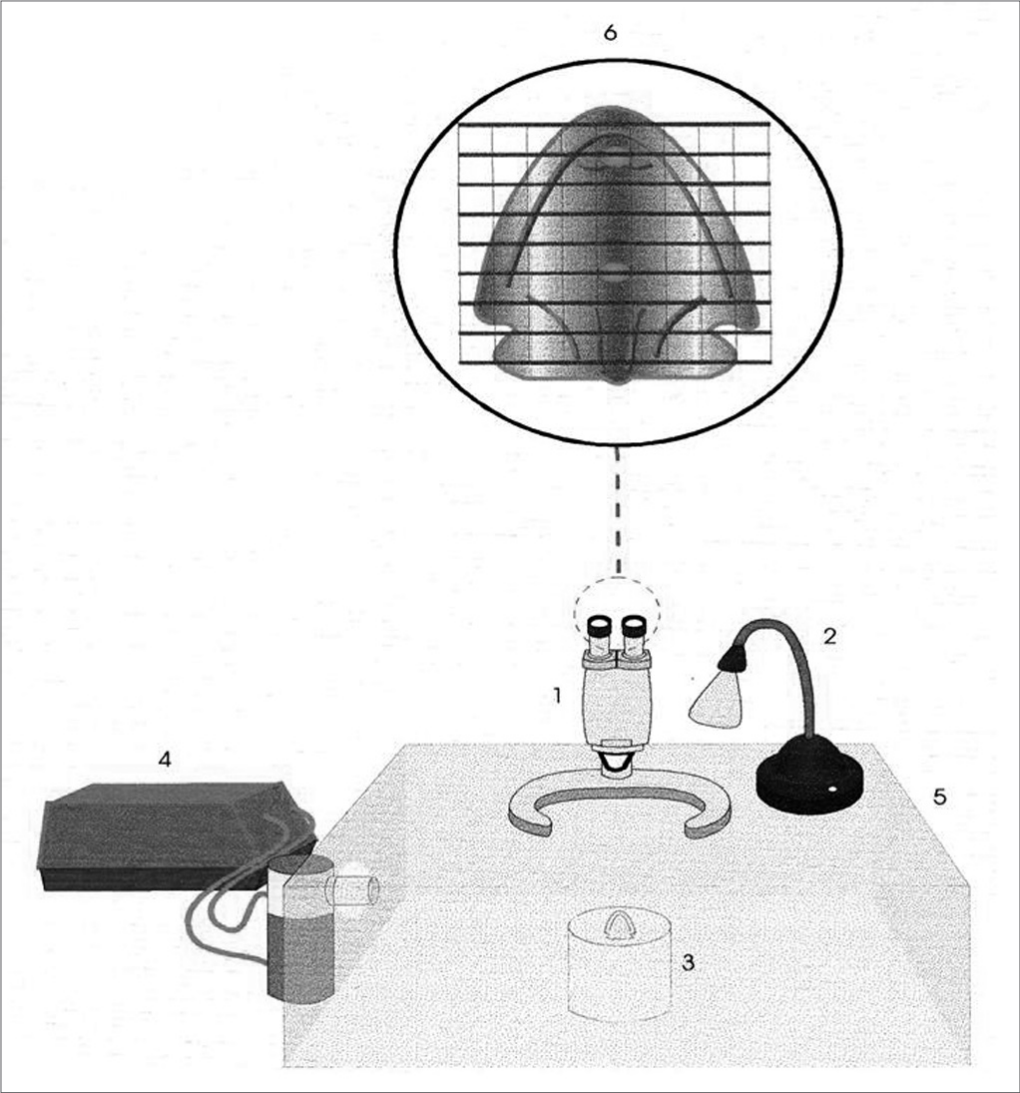
Schematic representation of the frog palate setup: 1) Magnifying glass, 2) Light source, 3) Frog palate support, 4) Ultrasonic nebulizer, 5) Acrylic chamber, 6) Frog palate vision under mashed lens. Source: Nakagawa et al., 2000.

Frog palate preparation is a convenient system to study the mucociliary transport and the mucus-cilia interaction (Figure 3). In their palate, frogs have a ciliated epithelium very similar to the one found in mammals, which we use to study the transportation method of different samples of mucus or ciliary activation or inhibition measures^1^.

The transportation velocity of a mucus sample placed on the frog palate’s smooth and flat surface is measured with the aid of a stereoscopic microscope with a meshed lens^21^, ^22^. The transportation velocity of the samples tested is later compared to the mucus of the frog itself, and the results are expressed in terms of relative velocity (mucus tested/frog’s mucus). The experiments are carried out under room temperature (20ºC), with the specimens kept in an acrylic chamber with 100% moist. Under such experimental conditions, the frog palate preparation is considered ideal, having as relevant variables only the mucus’s physical properties^1^.

### In vivo analysis of the mucociliary transport - Saccharin test

The first attempts to measure nasal mucociliary function in vivo were carried out by Martius in 1884 by means of stroboscopy. However, it did not attain much clinical applicability because of difficulties in handling the equipment.

In an attempt to simplify the mucociliary clearance analysis, Hilding^23^ and Tremble^24^ during the 30’s and 40’s respectively, introduced dyes in nasal cavities and measured how long it took to get to the oropharynx. Since it was not easy to visualize the dyes in many cases, in 1974 Andersen^25^ developed the saccharine test associated with food dyes, in order to increase the test sensitivity. In such setup, a small particle of sodium saccharine together with dye was placed behind the inferior turbinate head, and the patient would tell the examiner when he/she felt the sweet flavor or when the dye appeared in the oropharynx. Notwithstanding, since sodium saccharine is highly soluble in water, Deitmer26 stated that it could influence the composition of the periciliary fluid, thus altering the mucus clearance. In an attempt to counteract this error factor, an anionic resin was added to the saccharine, thus reducing its water solubility. In normal individuals, the test values varied from 3 min and 30s to 19 min and 15s, with a mean value of 10 min and 12.5s.

Because of its subjectiveness, more sophisticated tests were developed. In 1965, Proctor and Wagner^27^ used I131 as a marker and measure its movement through the nasal cavity through a gama-chamber, however, Harper^28^ noticed that Tecnecio 99m was the most adequate radioactive isotope used to calculate mucociliary transport.

### Transportation by cough and sneezing -Cough and sneezing simulator

In healthy individuals, mucus is primarily transported by cilia, however, in many pathologies where there is an overproduction of mucus, cough and sneezing clearance takes on a fundamental role.

Cough is characterized by inhaling approximately 2 litters of air, with a fast glottal closure and increase in pleural pressure to 100cm H_2_O or more. With glottal opening, there is a biphasic air flow expulsion with a fast initial component that lasts approximately 30-50ms followed by a slower component, which travels along the partially collapsed trachea. The expired gas hits the mucus layer and transfers part of its kinetic energy, moving the mucus towards the glottis^1^.

The in vitro analysis of transportation by cough and sneeze uses an equipment called “cough simulator”, adapted from King et al.^29^. It is made up of a compressed air reservoir which predicts air flow and is controlled by a solenoid valve, followed by a resistance which provides the turbulent flow - similar to that of cough and sneeze^1^.

**Figure 4:**
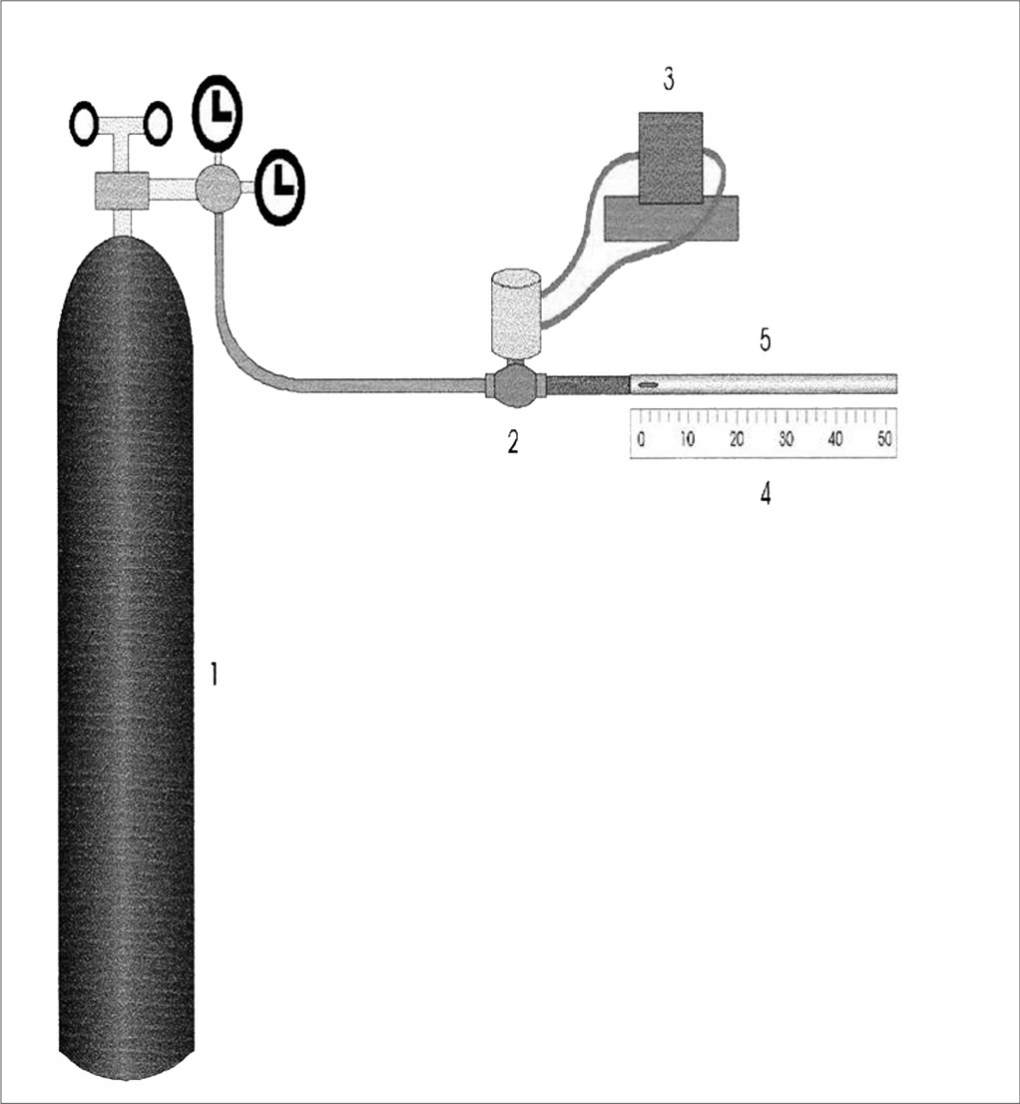
Schematic representation of the cough and sneezing simulator: 1) Compressed air reservoir, 2) Solenoid valve, 3) Solenoid valve controller, 4) Graded ruler, 5) Acrylic cylinder. Source: Nakagawa et al., 2000.

The trachea is simulated by an acrylic cylinder of 4mm of internal diameter and 133mm in length. The mucus sample is positioned with the help of a stylet at the entrance of the tube that is connected to the system, and its shifting is measured in millimeters with the help of a ruler (Figure 4).

## DISCUSSION

Mucociliary transport study and interpretation of the aforementioned tests require the understanding that the transport of secretions depend on three parameters:

1) Frequency and pattern of ciliary movement,
2) Viscoelastic and transportability properties of the respiratory mucus, and
3) mucus-cilia interaction1. Mucociliary transportation without the knowledge on these parameters may bring wrong conclusions and even inadequate treatments when the results obtained are extrapolated to the daily clinic.

Videoscopic and videomicroscopic methods used to study CBF use biopsy fragments or biopsies of respiratory epithelium cell smears, doing the measurement in an ex vivo fashion. The videoscopic technique is based on the principle that when the frequency of stroboscopic light is equal to that of CBF, one no longer sees the wavy movement on the epithelium surface. It is a reproducible method, however it depends on relatively subjective analysis, because measurement depends on personal observation^5^, ^6^. Videomicroscopy techniques assess CBF in a more precise way and aid in the assessment of frequency and ciliary movement patterns4. They require expensive and sophisticated equipment, still restricted to research protocols. They are techniques that do not assess CBF in vivo or in situ, they do not consider local factors and the characteristics of the organism to be evaluated as a whole.

The difficulties in analyzing the respiratory mucus already start at the collection phase. Obtaining bronchial secretions through bronchoscopes or tracheal aspiration tubes require invasive procedures and the need for specialized equipment and personnel, and also represent considerable morbidity to the patient. These difficulties made most studies use the mucus obtained through sputum, which is inconvenient because of the mixture of secretions with pharyngeal and oral cavity saliva; and also the difficulty in finding controls that usually do not have spontaneous mucus cough^1^.

Obtaining nasal mucus is easier, though it bears all the problems common to the lower airway such as the difficulty of collecting enough sample volume, especially from control subjects. Aspiration tubes require careful handling because high suction pressures may destroy mucus’s proteins and change the biologic behavior of samples. Cytology brushes may be an alternative, however they are usually obtained in small amounts.

In order to overcome the difficulties of studying mucus in humans, it is fundamental to work with animal models. By using proper techniques, Wistar rats may be used as an excellent animal model, since they are knowingly prone to developing inflammatory diseases of the airways, which is not seen in rats created under aseptic conditions (Specific Pathogen Free - SPF), allowing us to have a respiratory mucus study model under normal and pathological situations^30^.

Among the measures of respiratory mucus physical properties, the rheologic analysis is the most complete, because it provides information on sample viscoelasticity, which are variables that influence both in cilia transportation as well as in cough. Notwithstanding, rheologic analysis requires highly specialized personnel, because it is difficult to be performed, requires complex and difficult to handle equipment, and the analysis of a single sample may take hours to process. Moreover, it is very difficult to interpret the results and requires the understanding of fluid mechanics.

Contact angle measurement estimates how the mucus sample assessed will be transported by cough. Samples with low contact angle tend to be better transported by cough, with little influence in cilia clearance. Tandel 100 measure corresponds rheologically to the low contact angle, since samples with high values on these parameters have lower contact angle and more movement by cough^1^, ^14^.

Analysis of the respiratory mucus biochemical components have gained ground because of the presence and concentration of different elements, especially glycoproteins and immunoglobulins, directly influencing the rheologic and transportability characteristics of the samples. These observations make room for the development of new drugs for the treatment of over secretion of mucus and to better understand the pharmacologic actions of those already in the market^16^, ^17^, ^18^.

The difference in transepithelial electrical potential reflects the degree of respiratory epithelium integrity and has experimental applicability in the research of how aggressive factors such as air pollution or infectious agents interfere in its homeostasis. It bears reproductive results and simplicity in handling the equipment^1^.

Frog palate setup is an excellent method to study mucus transportation by the cilia because of the ease with which the technique is performed and the simplicity of the equipment used. Results analysis produce important information and offers an estimate of how certain mucus samples are transported by the cilia. Moreover, it bears close correlation with rheologic parameters, especially with LogG* 1 rad/s, which, according with Puchelle^22^, reflects the way through which the samples behave as they are transported by the cilia. However, it bears some limitations. It does not assess factors present in vivo that may influence cilia-mediated transport, such as anatomical alterations and periciliary fluid composition. In order to eliminate these biases, in vivo tests such as the saccharine, associated or not to dyes, and even those with radioisotopes did not represent solutions to the problems. They have a broad range of normality variability and depend on the patient’s subjective assessment, or expensive equipment of difficult clinical applicability. Despite the problems shown, they do have clinical applicability. They are used as screening in the investigation of cilliary dyskenisias, however with reduced sensitivity and specificity, and should always be associated with CBF analysis methods and those of ciliary axoneme ultra-structure for diagnostic confirmation^23^, ^24^, ^25^, ^26^, ^27^, ^28^.

The “cough and sneezing simulator”, used for the study of mucus transportation by high frequency stimuli is a simple method that has reproducible results. Besides informing how the mucus is tested and transported by cough and sneezing, it provides relevant estimates on the biologic behavior of these samples when submitted to the analysis of its physical properties. Cough-related transportation is positively correlated with the tandel 100 rad/sec rheological analysis and negative with the contact angle value. In other words, the higher the tandel 100 rad/s and lower the contact angle, more easily the mucus is transported by cough and sneezing^1^, ^29^.

## FINAL COMMENTS

Mucociliary transport dysfunctions reduce the life quality of patients, as in chronic rhinitis and sinusitis, all the way to severe consequences with the risk of developing irreversible and even fatal sequelae in the cases of cystic fibrosis and primary ciliary dyskinesias. Thus, when we have a patient we suspect of mucociliary clearance disorder, it is fundamental to understand the workings of the mucociliary apparatus and how changes in its components (cilia, periciliary fluid-mucus and the dynamic interaction between them) impact the transport of respiratory secretions

The methods mentioned in this review provide us with valuable information on the different aspects of mucociliary transport, and some of them are extremely easy to perform, and other are only applicable in research protocols because of technical difficulties and financial constraints. We also have to consider the lack of methods that assess CBF in vivo and in situ in an outpatient basis, which would enhance diagnoses of ciliary diskinesias, avoiding the need for more invasive procedures for diagnostic confirmation

There are still many questions to be answered on the disorders of mucociliary transport, however, the knowledge already acquired allow for a better understanding of how patients get sick and favor the institution of more rational treatment modalities in the disorders related to mucociliary transport.
